# Longitudinal processes among humility, social justice activism, transcendence, and well-being

**DOI:** 10.3389/fpsyg.2024.1332640

**Published:** 2024-03-08

**Authors:** Peter J. Jankowski, Steven J. Sandage, David C. Wang, Michael J. Zyphur, Sarah A. Crabtree, Elise J. Choe

**Affiliations:** ^1^Albert & Jessie Danielsen Institute, Boston University, Boston, MA, United States; ^2^Marriage and Family Therapy Program, Bethel University, Saint Paul, MN, United States; ^3^Fuller Theological Seminary, Pasadena, CA, United States; ^4^UQ Business School, Faculty of Business, Economics, & Law, University of Queensland, St. Lucia-Brisbane, QLD, Australia

**Keywords:** humility, activism, transcendence, well-being, longitudinal

## Abstract

**Introduction:**

Existing research shows positive associations between humility and well-being, and between civic engagement and well-being. Rarely have humility, civic engagement, and well-being been examined together. We build off of previous cross-sectional findings and a prior longitudinal study that used three waves of data and found significant positive bivariate correlations between humility and the presence of life purpose across time points.

**Methods:**

Extending these previous findings, we used six waves of data obtained from graduate students at 18 seminaries across North America (*N* = 574; *M_age_* = 31.54; 46.7% female; 65.3% White) to explore the dynamic associations among humility and life purpose, along with horizontal transcendence (an indicator of the attitudinal dimension of civic engagement) and social justice activism (an indicator for the behavioral dimension). We explored reciprocal short-run processes and dynamic long-run effects using a general cross-lagged panel model.

**Results and discussion:**

We found robust evidence for a reciprocal influence between the presence of life purpose and horizontal transcendence, and long-run effects for initial levels of life purpose to influence later levels of horizontal transcendence. We also found long-run effects for the influence of initial levels of life purpose on later levels of humility, and initial levels of social justice activism on later levels of horizontal transcendence. Implications center on the use of the findings for planning future one-time life purpose and social justice interventions to affect changes in humility and horizontal transcendence.

## Introduction

The construct of general humility, in part defined by other-oriented-ness, has demonstrated consistent positive associations with well-being (Davis et al., 2017a McElroy-Heltzel et al., 2019). Civic engagement, also defined by other-oriented-ness, has similarly shown positive associations with well-being, although these beneficial effects tend to vary by type of civic engagement, with some showing a nonsignificant influence (Wray-Lake et al., 2019 Fenn et al., 2021). In addition, some aspects of civic engagement may be detrimental to well-being (e.g., Chen and Gorski, 2015; Hill et al., 2023). Surprisingly, few studies have examined associations between humility and civic engagement, let alone examine these prosocial constructs together when predicting well-being. In fact, the scant existing findings are mixed. General humility has shown positive associations with attitudinal civic engagement (Jankowski et al., 2013; Bell et al., 2017), whereas intellectual humility, a derivative of general humility, has shown nonsignificant associations with behavioral civic engagement (Krumrei-Mancuso and Newman, 2020; Mcelroy-Heltzel et al., 2023). The attitudinal dimension refers to personal *beliefs* and *subjective experiences* oriented around improving others’ lives (e.g., awareness of injustice and concern for the welfare of others; Doolittle and Faul, 2013; Sanderson et al., 2019), whereas the behavioral dimension refers to *actions* explicitly geared toward improving others’ lives (Sanderson et al., 2019).

The existing findings are predominantly cross-sectional, a concern that has been frequently noted about the literature on humility and well-being (Davis et al., 2017a; McElroy-Heltzel et al., 2019). Cross-sectional findings may provide a glimpse of longitudinal processes (Zimmerman and Arunkumar, 1994), and yet, they offer limited causal conclusions because of an absence of temporal order (Zyphur et al., 2020a). In contrast, longitudinal designs that use observational data allow for *real-world effects* (Zyphur et al., 2020a). In the current study, we utilized the general cross-lagged panel model (GCLM; Zyphur et al., 2020a) to examine longitudinal associations between humility and eudaimonic well-being, the latter defined by the presence of life purpose, along with their relation to the attitudinal and behavioral dimensions of civic engagement. We used *horizontal transcendence,* defined as a subjective sense or experience of connection beyond-the-self to human others (King et al., 2017), as an indicator of the attitudinal dimension of civic engagement, and social justice activism, an indicator of the behavioral dimension (Pattie et al., 2003). As an attitude, horizontal transcendence consists of *values* (Barton and Hart, 2023), which we define as *the right or the good* to which individuals give importance (Schwartz, 2012; Fowers et al., 2021) and “beliefs about what is good, right, obligatory, and/or virtuous” (Tjeltveit, 2015, p. 36). Values can motivate action (Schwartz, 2012) and virtuousness (Fowers et al., 2021), with *virtuous* referring to exhibiting context relevant character strengths that constitute well-being (Fowers et al., 2021). Last, we examined these associations in a sample of emerging religious/spiritual (R/S) leaders for whom virtue development is part of their professional training and vocationally salient because of R/S leaders’ susceptibility to lower well-being (Jankowski et al., 2019, 2023). In addition, theological education reforms emphasize civic engagement competencies for emerging R/S leaders so they can better navigate the growing diversity and increased polarization in the congregations and the communities in which they are situated (Jankowski et al., 2022c).

### Distinguishing life purpose and horizontal transcendence

Before we map associations between humility, social justice activism, horizontal transcendence, and life purpose, we define life purpose and horizontal transcendence as distinct constructs. The need for greater conceptual clarity prior to empirical investigation has been increasingly recognized (e.g., Hodson, 2021; Lawson and Robins, 2021; Porter et al., 2022). This is especially the case for the constructs in the current study, which represent *sibling constructs* (Lawson and Robins, 2021) susceptible to the *jangle fallacy* (i.e., different labels but conceptually overlapping and empirically the same construct; Gonzalez et al., 2021). Life purpose is often conflated with meaning in life (Bronk and Mitchell, 2022; Bronk et al., 2023), and this conceptual ambiguity and redundancy is even more pronounced when purpose and meaning are conflated with transcendence, concerns we later extend to humility and transcendence. Transcendence can be defined broadly as “a beyond-the-self orientation” (Bronk et al., 2023, p. 1023), and we use the term *transcendence* in this broad sense, distinct from the more precise language, *horizontal transcendence*, which adds nuance to this broad definition.

Wong et al. (2021) referred to *life transcendence* as “an awareness of one’s worthy life purpose” (p. 8), largely synonymous with what Steger et al. (2006) referred to as *presence* of meaning in life, and Bronk et al. (2018) operationalized as the meaningfulness dimension of life purpose. Life purpose refers to personally meaningful life aims (Bronk and Mitchell, 2022), which connotes *subjective judgment* (Costin and Vignoles, 2020). Similarly, Steger et al. (2006) defined the presence of life purpose as “the subjective sense that one’s life is meaningful” (p. 85). In contrast, *transpersonal transcendence* is defined by an awareness of interpersonal connectedness (Wong et al., 2021). This awareness is infused throughout the other aspects of a beyond-the-self orientation: “mak[ing] a positive contribution to society,” virtuousness, and experiences of awe or elation *and* wonder which occur in response to an awareness of *vastness* (Wong et al., 2021, p. 6). We used the connectedness aspect of transpersonal transcendence to operationalize the attitudinal dimension of civic engagement as horizontal transcendence, defined as “a sense of relatedness …. [that is] experiential” (King et al., 2017, pp. 237, 246).

Wong et al. (2021) summary of Frankl’s theory posited the cognitive factor as identifying meaningful goals, which works in concert with the behavioral-motivational factor of aiming for those goals. Together, the two factors constitute life transcendence, which facilitates the development of transcendence. At the same time, transcendence “motivates individuals to live a meaningful life” (p. 6). Thus, implied in their description was a reciprocal process whereby life aims and goals interact over time with the elements comprising a beyond-the-self orientation. Ge and Yang (2023) explicitly acknowledged this reciprocity. They noted “bidirectional connections [between] the cognitive and motivational components” of life transcendence and transcendent experiences, social connectedness, and benevolent actions (p. 3). They further summarized reciprocity among these latter three aspects of transcendence. Experiences of awe may interact with “a drive for prosocial behaviors due to feelings of greater connectedness with the world” (p. 4).

The *making a positive contribution* aspect of transcendence has been operationalized as a dimension of life purpose (Bronk et al., 2018), and a response to valuing self-transcendence (i.e., “concern for the welfare and interests of others”) in contrast to the value of self-enhancement (i.e., “pursuit of one’s own interests … and dominance over others;” Schwartz, 2012, p. 8). Prior research has found that greater self-transcendence corresponded to greater activism, whereas greater self-enhancement corresponded to lower activism (Sanderson et al., 2019). We operationalized making a positive contribution as social justice activism, defined as “valuing diversity and challenging injustice and disparities in all its forms” (Leong et al., 2017, p. 779). King et al. (2017) similarly considered making a positive contribution to society to be a separate-yet-related dimension from that of transcendence. Their operationalization of transcendence included items that captured experienced connection with others. We used those items to operationalize horizontal transcendence in the current study.

In sum, *personally meaningful* judgments about one’s life aims can operationalize the presence of life purpose, which is distinct from a transcendent frame for life purpose based on aims beyond-the-self. We limit a beyond-the-self orientation to horizontal transcendence (i.e., the attitudinal dimension of civic engagement) *and* define making a positive contribution to the world as social justice activism (i.e., the behavioral dimension of civic engagement).

### Horizontal transcendence, humility, activism, and life purpose

Horizontal transcendence is differentiated from a vertical conceptualization which involves a sense of connection to the divine or sacred, and yet, they share the theme of beyond-the-self connection. Both aspects of transcendence can also be experienced as *spiritual* (Barton and Hart, 2023), in the sense of “being moved or called from within … [and] a burden of awareness” (McIntosh and Carmichael, 2019, p. 303). Barton and Hart (2023) noted that such *spiritual consciousness* often emerges out of adversity, or what others have defined as post-traumatic growth (Tedeschi et al., 2017). The intersection of transcendence, spiritual consciousness, post-traumatic growth, and social justice activism have been synthesized as *spiritual activism* (e.g., Keating, 2008; McIntosh and Carmichael, 2019).

In contrast, self-enhancement is the anti-thesis of transcendence (Schwartz, 2012) and as such taps into conceptualizations of narcissistic grandiosity (Jankowski et al., 2022a). We see humility as resisting self-enhancement (i.e., hypo-egoicism; Banker and Leary, 2020). As Bloomfield (2020) suggested, when “taking an inappropriately superior attitude toward another. Humility ought to then step in and check the problem” (p. 41). Humility seems to have particular relevance to the personal and professional development for those dedicated to making positive contributions to society (Wang et al., 2021), including emerging R/S leaders (Sandage et al., 2015). In fact, greater humility has demonstrated consistent associations with lower grandiosity in samples of R/S leaders (e.g., Jankowski et al., 2019, 2022a). Resisting self-enhancement involves *interpersonal openness* rather than self-superiority and a better-than-others relational stance (McElroy-Heltzel et al., 2019). Interpersonal openness represents a positive aspect of humility in contrast to the absence of negative aspects such as grandiosity and self-enhancement (Spezio et al., 2019). Operationally, interpersonal openness consists of valuing others’ influence and learning from them (Owens et al., 2013). These aspects connote healthy development, and are therefore the likely basis of the positive empirical associations between humility and well-being among seminary students (e.g., Jankowski et al., 2019; Ruffing et al., 2021).

Recent accounts about the relation between humility and social justice activism have been predominantly conceptual (e.g., Bloomfield, 2020; Wargin, 2022), with these accounts presenting conflicting perspectives. In one, humility is inversely associated with activism, and in the other, humility is positively associated with activism. Bloomfield (2020) suggested that “humility can be … a means of social control to maintain an unjust status quo.. Rebellion, however well-justified, is almost impossible in a climate too rich in humility” (p. 36). For Bloomfield, humility properly understood is a corrective to arrogance. Humility as *corrective self-restraint* can manifest as servility, that is, too much humility, and it is this sense in which humility can be an instrument of subjugation rather than a motivation for activism (Bloomfield, 2020). In contrast, Wargin (2022) argued that recasting humility within a transcendent, beyond-the-self, perspective allows humility to be positively associated with activism. Wright et al. (2018) described this as a low-self/high-other frame for humility, and suggested that “humility is the *experience of ‘all else’* … the vast web of interconnected beings whose needs/interests are … as worthy of attention and concern as one’s own” (p. 3). Wargin (2022) similarly suggested that this other-oriented transcendence perspective “renders one incapable of allowing one’s concern for one’s own comfort and security to stop one from pursuing justice” (p. 57).

However, such recasting of humility as transcendence raises concerns about construct proliferation whereby a construct becomes so multidimensional it overlaps with other constructs creating the construct redundancy problem (Hodson, 2021). Construct redundancy stems from developing new measures (Gonzalez et al., 2021) and a general inattention to discriminant validity (Lilienfeld and Strother, 2020). We define horizontal transcendence as *experienced connection* to others (King et al., 2017) distinct from an *interpersonal openness* frame for humility that resists self-enhancement (Spezio et al., 2019). Honesty-humility positively correlated with transcendence (Julian et al., 2021), and humility-as-transcendence demonstrated positive associations with social responsibility (Wright et al., 2018) and presence of life purpose (Davis et al., 2017b). However, we think it important to posit humility and transcendence as related-yet-distinct constructs. As Porter et al. (2022) suggested, researchers need to demonstrate clear distinctions among the multiple dimensions of a construct, and Lawson and Robins (2021) recommended *splitting* sibling constructs into their distinct dimensions and examine the dimensions as separate constructs.

Prior empirical research has found positive cross-sectional associations between interpersonal openness operationalizations of humility and social justice commitment (Jankowski et al., 2013; Bell et al., 2017), social justice commitment and transcendence, and social justice commitment and life purpose (Dy-Liacco et al., 2009). Hydinger et al. (2023) found positive associations between life purpose and social justice commitment, and life purpose and humility. C*ommitment* is an aspect of the attitudinal dimension of civic engagement and refers to the extent to which someone expresses concern about injustice (Baumert and Schmitt, 2016), whereas *activism* refers to actions that challenge injustice (Wargin, 2022) which can operationalize the behavioral dimension of civic engagement. As such, measures of commitment tend to have more items about an awareness of others’ unjust suffering, whereas measures of activism tend to reference engagement in specific behaviors to address injustice.

Prior research has also found significant longitudinal bivariate correlations between greater humility and greater presence of life purpose (Tong et al., 2019; Jankowski et al., 2022b). Tong et al. (2019) also found that greater psychological well-being, in part constituted by greater life purpose, longitudinally predicted greater humility. However, Jankowski et al. (2022b), using a smaller subset of the current study sample and three waves of data to test the influence of R/S exploration on well-being found a nonsignificant longitudinal predictive effect for humility on later life purpose. This finding is consistent with the nonsignificant longitudinal association between humility and psychological well-being found by Tong et al. (2019). In contrast, Jankowski et al. (2022e) used a larger sample than Jankowski et al. (2022b) and analyzed a latent variable panel model over three waves of data to test the influence of R/S commitment on well-being. Jankowski et al. (2022e) modeled lag-2 effects, which offered a greater degree of confound control (Lüdtke and Robitzsch, 2021), relative to the panel model of Jankowski et al. (2022b). Jankowski et al. (2022e) found that greater humility at time 2 predicted greater presence of life purpose at time 3, suggesting that interpersonal openness may foster personally meaningful judgments about one’s life aims. Alternatively, the overall mixed findings are consistent with Tong et al.’s (2019) conclusions that “psychological well-being could precipitate qualities associated with humility,” and conversely, “a humble person may not pursue important goals and succeed far enough to feel eudaimonic [well-being]” (p. 1355). The latter explanation for the nonsignificant effects seems consistent with theorizing about manifestations of humility as servility which inhibit the development of positive outcomes such as life purpose (Bloomfield, 2020).

## The current study

We hypothesized that (a) life purpose and horizontal transcendence would be reciprocally related over time (i.e., evidence of LP→TR *and* TR→LP “feedback’ or ‘reciprocal effects;’” in Zyphur et al., 2020a, p. 664; for a visual depiction of the GCLM using structural equation modeling notation see Zyphur et al., 2020a), and similarly, (b) horizontal transcendence and social justice activism would show a reciprocal association (i.e., TR→SJ *and* SJ→TR). Thus, while we expected reciprocity, we did not have expectations about which variables might be “causally dominant” or the “driving force” in the relations over time (Hamaker et al., 2015, p. 108). We simply expected greater presence of life purpose and greater sense of connection to others, and a greater sense of connection to others and greater active service for the good of others, to be mutually reinforcing.

Our first hypothesis was in part derived from theorized bidirectional relations between life and transpersonal transcendence (Wong et al., 2021 Ge and Yang, 2023). We reframed these constructs, respectively, as the subjective judgment about the presence of a meaningful life purpose and experienced connection with human others. Our expectation of reciprocity was also informed by longitudinal evidence showing a bidirectional association between presence of life purpose and collective connectedness (e.g., “I feel close to other people in my community;” Stavrova and Luhmann, 2016, p. 472). Collective connectedness connotes horizontal transcendence. Our second hypothesis is based on findings of a positive association between social justice commitment and transcendence (Dy-Liacco et al., 2009), transcendence and activism (Sanderson et al., 2019), and transcendence and making a positive contribution (King et al., 2017). In addition, Wray-Lake and Abrams (2020) noted reciprocity in the association between the attitudinal and behavioral dimensions of civic engagement, and Ge and Yang (2023) described reciprocity among the social connectedness and benevolent action elements of transcendence.

However, for humility, given mixed findings for longitudinal associations with life purpose (Tong et al., 2019; Jankowski et al., 2022b,e), and theorized mixed findings for associations with social justice activism (Bloomfield, 2020; Wargin, 2022), we did not have explicit hypotheses about these associations. However, we did expect humility to be positively associated with horizontal transcendence, given Wargin (2022) theorizing about an other-oriented transcendence framing for humility, and the finding of a positive association between life purpose and an operationalization of humility as transcendence (Davis et al., 2017a,b).

We tested these hypothesized associations in a longitudinal panel model. We modeled the associations among the conceptually distinct constructs of interpersonal openness, experienced connection to others, personally meaningful life aims, and activist behaviors over six waves of data using the GCLM. The various types of panel models share the strengths of modeling the temporal order among variables and bidirectional associations, whereas an additional strength of some panel models such as the GCLM is control of “stable between-unit differences” (Zyphur et al., 2020a, p. 658). We examined bidirectional relations as “short-run effects” within the GCLM by constraining the paths involved in the reciprocal exchange to zero, and assessed whether fit deteriorated relative to the unconstrained model (Zyphur et al., 2020a, p. 664). Another advantage of the GCLM is its usefulness in identifying entry points for targeting future interventions, based on a conceptualization of residuals as akin to how an actual intervention might influence the process among variables (Zyphur et al., 2020a; Shamsollahi et al., 2022). These “long-run effects” or *impulse responses* were examined by estimating the total effect of the residual on later variables (Zyphur et al., 2020a, p. 664). Last, we conducted a multigroup comparison to examine these associations under conditions of adversity, operationalized by interpersonal R/S conflict, given suggestions that life purpose, horizontal transcendence, and social justice activism may emerge out of or within such conditions (e.g., Wong et al., 2021 Barton and Hart, 2023; Ge and Yang, 2023). As such, we expected that the longitudinal associations among these variables may differ by level of adversity.

## Method

### Participants

As part of a larger study on the personal and professional development of emerging R/S leaders (e.g., Jankowski et al., 2022c,e,f, 2023), that utilized an “open cohort” design (Mahmud, 2010, para. 7), survey data were collected from graduate students at 18 seminaries across North America. The current study sample used the time 1 cohort (*N* = 574; *M_age_* = 31.50; *SD* = 11.12; range = 19–71) and six time points. A majority identified as male (52%; 47.3% female), heterosexual (92%; 2.5% gay/lesbian, 3.6% bisexual, 1.9% other), single/never married (54.4%), and as White (62.9%; 14.3% Asian, 8.7% Black, 6.0% Hispanic, 6.1% multiple races, 2.0% other). A majority attended evangelical Protestant affiliated seminaries (59%; 25.2% mainline Protestant, 11.9% Catholic, 3.8% Greek Orthodox). Wave 3 data, when we began assessing self-reported R/S identification showed that a majority identified as Christian (85%; 11.4% other; 3.1% none).

### Procedure

During the fall of 2019, students consented to complete an online survey, in exchange for a $25 gift card. The recruitment process was repeated approximately every 6 months, with wave 2 during spring of 2020 (*M* = 5.92 months later, *SD* = 1.24), wave 3 during fall 2020 (*M* = 6.84 months later, *SD* = 0.97), wave 4 during spring 2021 (*M* = 5.48 months later, *SD* = 0.82), wave 5 during fall 2021 (*M* = 6.75 months later, *SD* = 0.81), and wave 6 during spring 2022 (*M* = 5.52 months later, *SD* = 0.72). Seventy-six percent of the sample (*n* = 438) provided time 2 data, 80% (*n* = 460) provided time 3 data, 56% (*n* = 321) provided time 4 data, 43% (*n* = 246) provided time 5 data, and 38% (*n* = 219) provided time 6 data.

### Measures

#### Humility

We assessed humility using the 9-item Expressed Humility Scale (ω = 0.80 at time 1; e.g., “I am willing to learn from others;” Owens et al., 2013; rated 1 [*strongly disagree*] through 5 [*strongly*]). Higher mean scores indicated greater humility.

#### Horizontal transcendence

We used three items from the 11-item subscale of the Measure of Diverse Adolescent Spirituality (ω = 0.83 at time 1; e.g., “I feel that in some way my life is related with all of humankind;” King et al., 2017; rated 1 [*not true in my life*] to 5 [*almost always true in my life*]), with higher mean scores indicating greater sense of connection to human others.

#### Social justice activism

We used five items (ω = 0.91 at time 1; e.g., “I speak out for equality for immigrant communities;” Choi et al., 2019; rated 1 [*never true*] through 5 [*always true*]), with higher mean scores indicating greater social justice activism.

#### Life purpose

We used the four meaningfulness items from the Claremont Purpose Scale (ω = 0.90 at time 1; e.g., “How clear is your sense of purpose in your life?” Bronk et al., 2018; rated 1 [*not at all clear*] to 5 [*extremely clear*]), with higher mean scores indicating greater presence of life purpose.

#### Religious/spiritual conflict

We used two items from the Religious and Spiritual Struggles Scale (α = 0.75 at time 1; e.g., “felt hurt, mistreated, or offended by religious/spiritual people” and “had conflicts with other people about religious/spiritual matters;” Exline et al., 2014). Items were rated 1 (*not at all/does not apply*) to 5 (*a great deal*). Higher scores indicated greater interpersonal R/S conflict.

### Data analysis

Longitudinal data were analyzed using Mplus (version 8.4; Muthén and Muthén, 1998–2019; estimation = maximum likelihood estimation with robust standard errors or ‘MLR’). Data were multivariate non-normal (Mardia’s multivariate kurtosis statistic = 17.75, *p* < 0.001). Data were assumed to be missing at random (MAR) based on the low normed χ^2^ (χ^2^/df) of 1.11 for the Little’s MCAR test. The normed χ^2^ is a frequently used criterion for assessing the missing data mechanism in longitudinal designs (e.g., Nelemans et al., 2020; Becht et al., 2021). We therefore handled missing data using full-information maximum likelihood estimation and Bayesian analysis, methods which estimate each parameter using all available data (Muthén, 2016). We also conducted an attrition analysis using logistic regression. The five study variables and demographic variables at time 1 were modeled as predictors of the dichotomous variable comprising those with complete data across all six time points and those who did not provide data for times 2–6. Age was the only significant predictor of incomplete data (*B* = 0.05, *SE* = 0.02, 95% confidence interval [CI; 0.02, 0.08]; 2,000 bias-corrected bootstrap samples for the maximum likelihood estimation of the model). Older participants were more likely to have missing data.

We followed published guidelines to adopt a model-building and model-comparison approach (Zyphur et al., 2020a; Muthén, 2021; Shamsollahi et al., 2022). We used model fit indices (i.e., root mean square error of approximation [RMSEA], comparative fit index [CFI]), the Akaike information criterion (AIC) and the Bayesian information criterion (BIC) to compare models (Zyphur et al., 2020a; Muthén, 2021). With the traditional cross-lagged panel model as a starting point, we then added unit effects (i.e., ‘fixed effects’, ‘random intercepts’, or stable between-subject factors) and moving averages within the frameworks of the GCLM (Zyphur et al., 2020a) and residual structural equation modeling (Asparouhov and Muthén, 2023). The GCLM is distinguished by freely estimating, at a minimum, the first factor loading for the unit effect (Zyphur et al., 2020a; Martin and Zyphur, 2022).

The GCLM is a dynamic model, where ‘dynamic’ means that the past influences the future in a model (Muthén, 2021). Unit effects control for potentially confounding stable factors (Zyphur et al., 2020a; Shamsollahi et al., 2022), and the influence of a previous shock or ‘impulse’ on the future is modeled by a moving average (MA; Asparouhov and Muthén, 2023). The impulse is a random term operationalized as a residual, which represents “an unpredictable ‘shock’ or ‘innovation’ that is meant to mimic what may happen due to an intervention” (Shamsollahi et al., 2022, p. 5), and the MAs carry forward this “unpredictable ‘surprise’ in the system being modeled over time” (Zyphur et al., 2020a, p. 658). Strengths of the GCLM include modeling the temporal order among variables, reciprocal processes (i.e., short-run effects), the simultaneous influence of all autoregressive (AR), cross-lagged (CL), and MA and CLMA effects over time (i.e., long-run effects), and automatically controlling for potential confounders (Zyphur et al., 2020a,b).

## Results

Table 1 presents the latent factor correlations showing evidence of discriminant validity among constructs, and specifically, that the 95% bootstrap confidence interval did not cover 0.80 (Rönkkö and Cho, 2022). With the traditional cross-lagged panel model of associations between humility, horizontal transcendence, social justice activism, and life purpose modeled over six waves of data as a foundation, we then proceeded with the model building and comparison process. The results are presented in Table 2. Models 10 and 12 emerged as candidate models, with model 12 favored by the lowest AIC, which “favors the more complex” model relative to the BIC which favors the simpler model (Zyphur et al., 2020a, p. 672). Model 10 was favored by the next lowest AIC and lower BIC relative to model 12. Both models afforded substantive interpretation of the modeled processes (Zyphur et al., 2020a).

**Table 1:** Sample latent factor correlations at Time 1.

	Humility	Transcendence	Life purpose	SJA
Humility	--	0.29 (0.18, 0.39)	0.24 (0.13, 0.35)	0.24 (0.14, 0.35)
Transcendence		--	0.18 (0.08, 0.27)	0.33 (0.23, 0.42)
Life Purpose			--	0.14 (0.04, 0.23)
SJA				--

SJA, social justice activism. Percentile bootstrap 95% confidence interval in parentheses, using 5,000 bootstrap samples.

**Table 2:** Model fit statistics for the frequentist model comparison process.

Model	RMSEA (*p* < 0.05)	CFI	AIC	BIC
1. D-AR1-CL1	0.082 (0.00)	0.872	14313.79	15027.62
2. D-AR2-CL2	0.052 (0.356)	0.970	13909.15	14901.55
3. D-AR2-CL1	0.047 (0.796)	0.963	13892.92	14676.39
4. D-RI-AR1-CL1	0.029 (1.00)	0.985	13782.79	14540.15
5. D-RI-AR1-CL1 time noninvariant	0.029 (1.00)	0.979	13749.09	14227.87
6. D-RI-AR1-CL1 GCLM	0.025 (1.00)	0.986	13733.52	14299.36
7. D-RI-AR2-CL1	0.029 (1.00)	0.987	13790.91	14617.91
8. D-RI-AR2-CL2	0.029 (1.00)	0.992	13813.73	14849.65
^+^9. D-RI-AR1-CL1-MA1	0.026 (1.00)	0.984	13728.54	14224.74
10. D-RI-AR1-CL1-MA1 GCLM*	0.023 (1.00)	0.988	13712.87	14226.48
11. D-RI-AR1-CL1-MA1-CLMA1	0.025 (1.00)	0.986	13729.02	14277.45
12. D-RI-AR1-CL1-MA1-CLMA1 GCLM**	0.020 (1.00)	0.991	13709.60	14275.44
13. RI-CLPM	0.025 (1.00)	0.989	13763.11	14520.47
14. RI-CLPM time noninvariant	0.027 (1.00)	0.982	13735.16	14213.95

*N* = 574. RMSEA, root mean square error of approximation; p, probability that the RMSEA is below 0.05, RMSEA < 0.06 = good fit (Schreiber et al., 2006), RMSEA < 0.08 = acceptable fit (Brown, 2006), CFI, comparative fit index, CFI > 0.95 = good fit (Schreiber et al., 2006), CFI > 0.90 = acceptable fit (Brown, 2006); AIC, Akaike information criterion; BIC, Bayesian Information Criterion; AR1, autoregressive effect with 1-unit time-lag; AR2, autoregressive effect with 2-unit time-lag; CL1, cross-lagged effect with 1-unit time-lag; CL2, cross-lagged effect with 2-unit time-lag; GCLM, general cross-lagged panel model with indicators for the unit effect freely estimated, except for those set to 1 to obtain identification (Zyphur et al., 2020a); MA1, moving average effect with 1-unit time-lag; CLMA1, cross-lagged moving average effect with 1-unit time-lag. ^+^ With six time points, the introduction of moving averages is best modeled as time invariant (Asparouhov and Muthén, 2023); * “mean stability assumption by restricting the factor loadings to equality over time (after the t = 1 occasion …)” (Zyphur et al., 2021, p. 8); ** first loadings free for identification (Martin and Zyphur, 2022).

However, results also highlighted identification difficulties for models with moving averages and freely estimated factor loadings for the unit effect. Specifically, model identification was not achieved for GCLMs in which the time 6 factor loading was set to 1, and the loadings for times 1–5 were freely estimated, nor was identification achieved for model 10 when only the first factor loading was feely estimated and the loadings for *t* > 1 were set to 1. For model 12, tests of the long-run effects using bootstrapping would not converge (e.g., Martin and Zyphur, 2022). As Zyphur et al. (2021) noted, maximum likelihood estimation can have difficulty “when estimating time-varying unit effects and multiple lagged effects” (p. 2), whereas Bayesian estimation can fit the models. Such a use of Bayesian analysis is consistent with *computational frequentism* (Levy and McNeish, 2023), which involves “turn[ing] to Bayesian methods to bypass complexities posed by frequentist methods” (p. 721).

Zyphur et al. (2021) further noted that choosing the parameterization for the unit effect factor loadings is arbitrary, with alternatives necessary to obtain model identification. Therefore, as a next step, we compared three variations each for models 10 and 12 with alternative parameterizations for the unit effects using Bayesian estimation. The Gelman-Rubin criterion assessed convergence, with a potential scale reduction factor (PSR) < 1.10, and ideally “not much larger than 1” (Muthén and Asparouhov, 2012, p. 335). We then re-ran the models using a large fixed number of iterations to ensure stabilization of the PSR < 1.10 (Muthén and Asparouhov, 2012). Model fit was evaluated using the posterior predictive *p*-value (*PPP*). A *PPP* > 0.05 indicates acceptable fit, and “values between 0.05 and 0.20 … considered approximately fitting” (Asparouhov and Muthén, 2021, p. 6). A *PPP* around 0.50 indicates excellent fit (Muthén and Asparouhov, 2012). A better-fitting model is also indicated by a symmetric confidence interval around zero for the difference between the observed and the replicated chi-square values (Muthén and Asparouhov, 2012, p. 315). Model comparison also involved the Deviance Information Criterion (DIC), which is the “Bayesian analog of AIC” (Spiegelhalter et al., 2002, p. 613), with a lower value indicating better fit.

Table 3 presents the results of the model building and comparison process. We used the default noninformative or diffuse priors in Mplus, except where we note alternatives, and we used the default percentile-base credibility intervals for the CI around the modeled effects (Muthén and Muthén, 1998–2019). As an initial step to fit the models, impulse variances were set to 0.005 to aid convergence (Zyphur et al., 2021) and we modeled small-variance priors for differences between AR, CL, MA, and CLMA effects, which operationalized the assumption “that the parameters are similar over time” (Zyphur et al., 2021, p. 8). Models 1 and 4 represent the GCLM with the time 1 factor loading set to 1 and estimated loadings with small-variance priors for differences between loadings *t* > 1 [DIFFERENCE(λ2-λ6) ~ *N*(0.00,0.01)] to operationalize mean stationarity over time (Zyphur et al., 2021). In general, such small-variance priors and small-variance prior differences are used to improve model parsimony by approximately fixed parameters to some value or equality, while also allowing the observed data to influence model estimates when such priors are not justified empirically.

**Table 3:** Model fit statistics for the Bayesian model comparison process.

Model	*PPP*	95% CI	DIC
1. D-RI-AR1-CL1-MA1 GCLM time noninvariant	0.41	−71.32, 86.18	13714.91
2. D-RI-AR1-CL1-MA1 GCLM time noninvariant no effect	0.22	−49.39, 109.06	13734.45
3. D-RI-AR1-CL1-MA1 GCLM time noninvariant informed	0.44	−73.81, 83.51	13716.62
4. D-RI-AR1-CL1-MA1-CLMA1 GCLM time noninvariant	0.64	−95.49, 64.65	13715.72
5. D-RI-AR1-CL1-MA1-CLMA1 GCLM time noninvariant no effect	0.58	−89.80, 72.39	13722.66
6. D-RI-AR1-CL1-MA1-CLMA1 GCLM time noninvariant informed	0.65	−98.06, 63.64	13714.14

*N* = 574. 200,000 Markov chain Monte Carlo analysis samples; GCLM time noninvariant, unit effect time 1 loading set to 1, and times 2–6 set to small-variance priors using DIFFERENCE(λ2-λ6) ~ *N*(0.00,0.01) and small-variance priors for differences in time-varying associations among manifest variables; GCLM no effect, unit effect time 1 loading set to 1, and times 2–6 set with small variance priors, e.g., λ2 ~ *N*(0.00,0.01) … λ6 ~ N(0.00,0.01) and small-variance priors for differences in time-varying associations among manifest variables; GCLM informed, stationarity operationalization for humility and horizontal transcendence unit effects and no effect operationalization for life purpose and social justice activism, and small-variance priors for differences in time-varying associations among manifest variables; PPP, posterior predictive *p*-value; 95% CI, 95% Confidence Interval for the difference between the observed and the replicated chi-square values; DIC, deviance information criterion.

Models 2 and 5 represent the GCLM with the time 1 factor loading set to 1 and estimated loadings with small-variance priors for *t* > 1 [e.g., λ2 ~ *N*(0.00,0.01) … λ6 ~ *N*(0.00,0.01)], which operationalizes the assumption of “no unit effects” for *t* > 1 (Zyphur et al., 2021, p. 8). Models 3 and 6 represent different assumptions for the unit effects, (a) “constant effects” for humility and transcendence, and (b) “small if any unit effects” for life purpose and social justice activism (Zyphur et al., 2021, p. 8). Models 3 and 6 were informed by results that applied uniform constant and no effects assumptions to the factor loadings in prior tests of the models. We opted to interpret Model 3 based on DIC values which showed no appreciable improvement in model fit with the addition of CLMA terms, overall fit, and substantive interpretability (for an approximate visual depiction of Model 3 see Figure 3 in Zyphur et al., 2020a).

Short-run effects were examined using unconstrained versus constrained model comparisons, with CL paths fixed to zero for the constrained model, which implemented “Granger-Sims causality tests” (Zyphur et al., 2020a, p. 664). A deterioration in fit was assessed using information criterion (Zyphur, 2019) to determine reciprocal dynamics between variables (Zyphur et al., 2020a). We used ΔDIC (Asparouhov et al., 2018; Zyphur et al., 2021) and a criterion of Δ > 3 (Cain and Zhang, 2019) to indicate a potentially meaningful difference between models. We found evidence of reciprocity as the constrained paths from LP→TR and TR→LP showed a deterioration in fit (ΔDIC = 13.00).

**Figure 1:**
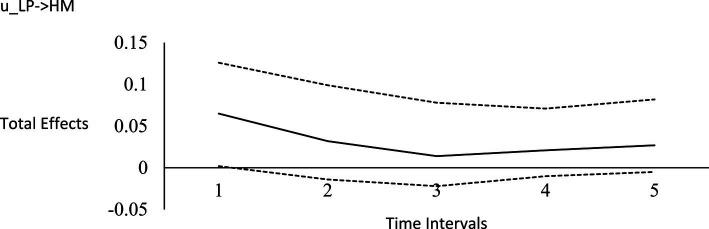
Impulse response function for life purpose on humility. Unstandardized effects and 95% confidence intervals. Plot depicts the influence of the initial random impulse on the outcome over time. A level plot indicates a persistence process, whereas a slope trending to zero indicates a “mean-reverting process” (Zyphur et al., 2020a, p. 669). u_LP, random impulse for life purpose; HM, humility. Time intervals, the effect of the initial level on the variable at time 1 on subsequent levels of that variable, time points 2–6.

**Figure 2:**
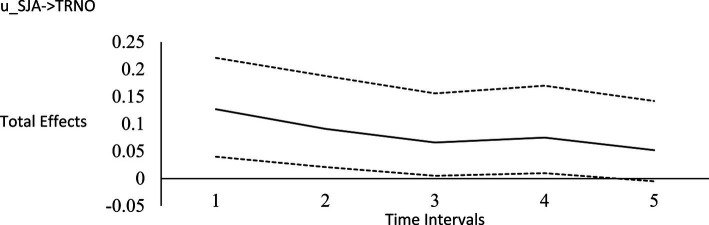
Impulse response function for social justice activism on horizontal transcendence. Unstandardized effects and 95% confidence intervals. Plot depicts the influence of the initial random impulse on the outcome over time. A level plot indicates a persistence process, whereas a slope trending to zero indicates a “mean-reverting process” (Zyphur et al., 2020a, p. 669). u_SJA, random impulse for social justice activism; TRNO, horizontal transcendence; Time intervals, the effect of the initial level on the variable at time 1 on subsequent levels of that variable, time points 2–6.

**Figure 3:**
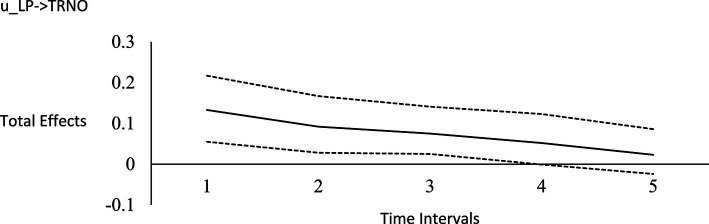
Impulse response function for life purpose on horizontal transcendence. Unstandardized effects and 95% confidence intervals. Plot depicts the influence of the initial random impulse on the outcome over time. A level plot indicates a persistence process, whereas a slope trending to zero indicates a “mean-reverting process” (Zyphur et al., 2020a, p. 669). u_LP, random impulse for life purpose; TRNO, horizontal transcendence. Time intervals, the effect of the initial level on the variable at time 1 on subsequent levels of that variable, time points 2–6.

**Figure 4:**
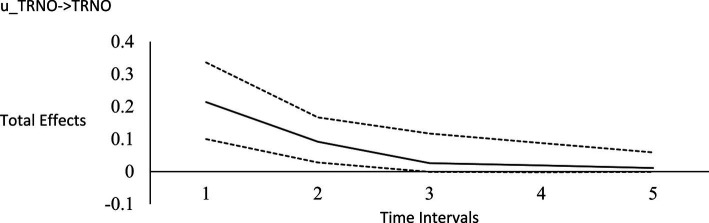
Impulse response function for horizontal transcendence. Unstandardized effects and 95% confidence intervals. Plot depicts the influence of the initial random impulse on the outcome over time. A level plot indicates a persistence process, whereas a slope trending to zero indicates a “mean-reverting process” (Zyphur et al., 2020a, p. 669). u_TRNO, random impulse for horizontal transcendence. Time intervals, the effect of the initial level on the variable at time 1 on subsequent levels of that variable, time points 2–6.

**Figure 5:**
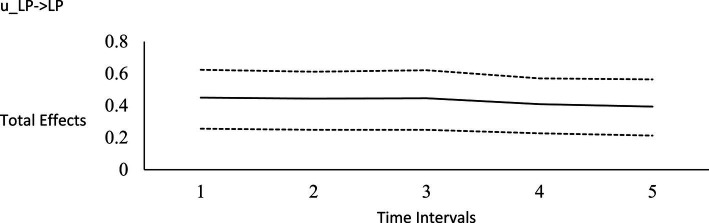
Impulse response function for life purpose. Unstandardized effects and 95% confidence intervals. Plot depicts the influence of the initial random impulse on the outcome over time. A level plot indicates a persistence process, whereas a slope trending to zero indicates a “mean-reverting process” (Zyphur et al., 2020a, p. 669). u_LP, random impulse for life purpose. Time intervals, the effect of the initial level on the variable at time 1 on subsequent levels of that variable, time points 2–6.

**Figure 6:**
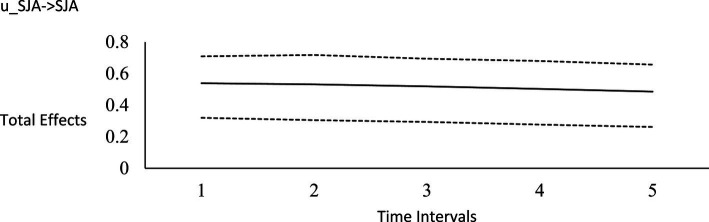
Impulse response function for social justice activism. Unstandardized effects and 95% confidence intervals. Plot depicts the influence of the initial random impulse on the outcome over time. A level plot indicates a persistence process, whereas a slope trending to zero indicates a “mean-reverting process” (Zyphur et al., 2020a, p. 669). u_SJA, random impulse for social justice activism. Time intervals, the effect of the initial level on the variable at time 1 on subsequent levels of that variable, time points 2–6.

For the long-run effects, we employed Bayesian estimation as a bootstrapping analog to generate 95% credibility intervals for the total effects (Zyphur et al., 2020a). This use of Bayesian analysis is also consistent with *computational frequentism* as noted previously. Long-run effects are computed by tracing the influence of the random impulse along the paths throughout the model. Figures 1–6 display the impulse response functions which plot the total effects (generated using the Excel worksheet from Zyphur et al., 2020a) tracing MA, AR and CL paths. Significant associations are depicted by a credibility interval that does not cover zero, and are shown in Figures 1–6. Figures 1–4 depict effects which trend toward zero over time, or mean-reversion processes, whereas Figures 5, 6 display persistent effects over time. Of note, the long-run effects for humility on subsequent levels of humility were nonsignificant.

Plateaus in which the credibility intervals do not cover zero suggest that a one-time intervention may have a more prolonged influence, whereas a mean-reversion process connotes that the influence of a one-time intervention is of shorter duration. As Zyphur et al. (2020a) noted, “plots of the effects offer a simple way to see how interventions may work” (p. 665). Results suggest that an initial perturbation on life purpose may have a somewhat persistent influence on later levels of transcendence, becoming nonsignificant during the fourth interval (i.e., by time point 5), whereas the effect for social justice activism may persist a little further into the future to influence later levels of transcendence, becoming nonsignificant during the fifth interval (i.e., by time point 6). The influence of an initial perturbation on life purpose has a shorter-run influence on later levels of humility, becoming nonsignificant by the second time interval (i.e., by time point 3 in a time 1 → time 3 effect).

### Multigroup comparison

We examined whether the D-RI-AR1-CL1-MA1 GCLM differed by initial levels of interpersonal conflict by conducting unconstrained versus constrained model comparisons of factor loadings, AR, CL, and MA parameters. We created a dichotomous variable using a median split (Iacobucci et al., 2015) that showed a significant difference between groups on adversity [*t* = −33.88(572), *p* < 0.001; *d* = 1.31]. The dichotomous variable reflected low (coded 0; *n* = 266; *M* = 2.92, *SD* = 0.84) and high interpersonal conflict (coded 1; *n* = 308; *M* = 6.63, *SD* = 1.60). In the context of multigroup analysis, the parameter restrictions required to estimate the model, including the pattern of impulse (unit) covariances, proved problematic for the Bayes estimator. As a result, we estimated the model using MLR, with the GROUPING option, and the MODEL TEST command (i.e., the Wald test of parameter constraints; Asparouhov and Muthén, 2021; Muthén and Muthén, 1998–2019). We modeled a mean stability assumption for the factor loadings (i.e., time 1 loading set to 1, and estimated loadings for times 2–6 set to equivalent within each process; Zyphur et al., 2021) and time invariant path coefficients; that is, we specified Model 10 from Table 2. These specifications modeled the assumption that parameters would be similar over time, consistent with the use of small-variance priors described above and the GCLM more generally (Zyphur et al., 2021). The comparison yielded no difference between unconstrained and constrained models [Wald χ^2^ = 34.30(24), *p* = 0.08]. As a follow-up to the attrition analysis, we used a “saturated correlates” approach to inform model estimation in the presence of missing data that are dependent on age (Asparouhov and Muthén, 2008, p. 2; Muthén, 2020). The comparison again yielded no difference between models [Wald *χ*^2^ = 32.88(24), *p* = 0.11]. These results suggest that the process among variables modeled by factor loadings, AR, CL, and MA parameters did not differ between those reporting low levels of interpersonal R/S conflict relative to those reporting high levels of conflict.

### Sensitivity analysis

Best practice recommendations for statistical modeling call for a sensitivity analysis, which involves “running alternative, justifiable analyses to see whether a reported result would still hold up” (Nuijten, 2022, p. 392). We selected the random intercept cross-lagged panel model (RI-CLPM; model 14 from Table 2) for the sensitivity analysis because the model had the lowest BIC. We also used the RI-CLPM because of its prevalence and familiarity (Mulder and Hamaker, 2021; Usami, 2021).

**Figure 7:**
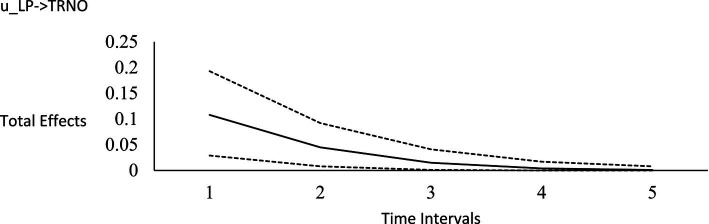
Long-run effect for life purpose on horizontal transcendence from the RI-CLPM. Unstandardized effects and 95% confidence intervals. Plot depicts the within level process for life purpose to influence transcendence over time. A level plot indicates a persistence process, whereas a slope trending to zero indicates a “mean-reverting process” (Zyphur et al., 2020a, p. 669). LP, life purpose; TRNO, horizontal transcendence. Time intervals, the effect of the initial level on the variable at time 1 on subsequent levels of that variable, time points 2–6.

Results from the tests for short-run effects revealed a reciprocal process between life purpose and horizontal transcendence (ΔAIC = 5.39). For the long-run effects, we used bootstrapping to generate 95% confidence intervals for the total effects (Zyphur et al., 2020a). Figure 7 depicts a mean-reversion process in which initial levels of life purpose influenced later horizontal transcendence. These results provide evidence for the robustness of the life purpose – horizontal transcendence association.

One reason why the other long-run effects were not replicated may be due to the absence of MA terms, which limit the complexity of the processes that can be modeled in the RI-CLPM. In terms of the moving averages, as Zyphur et al. (2020a) noted, “the dynamic process linking the past and the future via AR and CL terms is assumed to follow a simple, indirect-effects structure,” whereas MAs allow for discerning how the past may “persist (or fade) in complex ways” (p. 658). MAs also “modify AR [and CL] paths by making observations a direct function of past impulses” (p. 660). Furthermore, the RI-CLPM imposes an assumption of mean stationarity in a process from the first occasion onwards, which is very different from the GCLM which makes fewer assumptions about the stability of a process (Zyphur et al., 2020b). The difference is that the RI-CLPM can ‘soak up’ observed covariance among variables over time, which can lead to a lack of significant over-time effects in a model even if they are present in the real-world phenomena being modeled.

We then conducted multigroup analysis comparing the unconstrained versus constrained models (Mulder and Hamaker, 2021). We used the GROUPING command (Muthén and Muthén, 1998–2019). The comparison yielded no difference between models (maximum likelihood robust Δχ^2^ = 7.42(16), *p* = 0.96; ΔCFI = 0.004; ΔRMSEA = 0.002; i.e., no difference = nonsignificant Δχ^2^, Mulder and Hamaker, 2021; ΔCFI <0.01, ΔRMSEA <0.01, Putnick and Bornstein, 2016). Model 14 from Table 2 did not differ between those reporting low relative to those reporting high levels of interpersonal R/S conflict.

## Discussion

Consistent with hypothesized associations, we found that life purpose and horizontal transcendence showed a positive reciprocal dynamic over time. As theorized above, both presence of life purpose and horizontal transcendence can be motivational, and both forward looking life aims and experienced connection to others can be judged as personally meaningful, and therefore mutually reinforcing. We also found longitudinal associations showing that initial levels of greater life purpose predicted later levels of greater horizontal transcendence *and* greater humility, and that initial levels of greater social justice activism predicted greater horizontal transcendence later. This long-run effect for life purpose on horizontal transcendence is consistent with Wong et al.’s (2021) summary of Frankl’s two-factor theory which posited presence of life purpose as a precipitator of transpersonal transcendence, although as noted above there is a reciprocity such that life and transpersonal transcendence may mutually foster the other. Long-run effects were also consistent with the notion that greater life purpose may “precipitate qualities associated with humility” (Tong et al., 2019, p. 1355), that is, personally meaningful judgments about greater presence of life purpose seems to foster greater interpersonal openness. The current finding of a life purpose → humility association potentially clarifies prior inconsistent longitudinal findings, including studies with seminary students (e.g., Jankowski et al., 2022b,e), because the current modeling approach controlled for stable between-subject factors by including unit effects.

In contrast, and contrary to expectations, we did not find reciprocal relations between the attitudinal and behavioral dimensions of civic engagement (Wray-Lake and Abrams, 2020), rather we found that social justice activism predicted horizontal transcendence. This finding is consistent with the virtue ethics proposition that engagement in virtuous behavior can promote greater virtuousness (Jankowski et al., 2020), and in this case, the virtue of transcendence (Wong et al., 2021), and specifically, horizontal transcendence. This finding is also consistent with a multi-dimensional conceptualization for transcendence (King et al., 2017 Wong et al., 2021). However, rather than conflating the connection to others with contributing to society, we operationalized these dimensions as sufficiently distinct, *and* distinct from personally meaningful judgments about the presence of life purpose. Multi-dimensional constructs can benefit from “a clear distinction when measuring [their] unique features” which then permits examining “how the distinctive features relate to and shape one another” and relate to external correlates (Porter et al., 2022, p. 525). Such an approach is necessary to avoid jingle-jangle fallacies (i.e., mis-conceptualizing distinct constructs as the same and mis-conceptualizing the same constructs as distinct; Lilienfeld and Strother, 2020; Gonzalez et al., 2021; Hodson, 2021; Porter et al., 2022). As we show below, conceptualizing and modeling these aspects of transcendence as distinct holds greater usefulness for planning interventions. Also contrary to expectations, we did not find support for the other-oriented transcendence perspective of humility (Wargin, 2022). We observed nonsignificant associations between humility and horizontal transcendence, and between humility and social justice activism. Last, contrary to expectations we found that the modeled associations did not differ by low relative to high interpersonal R/S conflict.

### Practical implications

One of the advantages of the modeling strategy we employed is its utility for conceptualizing and identifying entry points for future interventions (Zyphur et al., 2020a; Shamsollahi et al., 2022). Results from the tests of the long-run effects suggested that a one-time intervention designed to target life purpose or social justice activism may affect gains in horizontal transcendence. Life purpose interventions constitute a broad class of interventions, which have shown effectiveness at improving personally meaningful judgments about one’s life, along with improved well-being and symptom outcomes (e.g., Guerrero-Torrelles et al., 2017; Park et al., 2019; Manco and Hamby, 2021). While the interventions employ diverse strategies to facilitate meaning-making amidst individuals’ lived experience, Manco and Hamby (2021) found that the meta-analytic evidence favored interventions grounded in mindfulness and narrative reconstruction.

Social justice activism interventions, or more broadly, prejudice reduction strategies, seem to primarily center on direct and indirect intergroup contact approaches, with direct contact approaches showing a long research history of beneficial effects (Stefaniak et al., 2022). Recent attention however has focused on the contraindications for direct contact approaches, and subsequently the notion that indirect approaches should be an initial step to changing attitudes toward diverse others. Stefaniak et al. (2022) offered an indirect approach founded on psychoeducation about the in-and outgroups’ *shared history* of a geographic location. They found evidence that the intervention promoted gains in direct contact intentions, perspective-taking, place attachment, and civic engagement intentions.

Jankowski et al. (2022c) offered a summary of social justice interventions around themes of emotional engagement and regulation. They noted that for many individuals from dominant groups, changes to the social order can be perceived as threatening to their identity constructions with accompanying fear, anxiety, and/or anger. Mindfulness and reflective practices inherent to contemplative pedagogy can foster affect regulation and prosociality. In fact, mindfulness mediated positive associations between emotion regulation and humility, and reflective functioning and humility, among seminary students (Captari et al., 2021). However, empirical evidence for the effectiveness of contemplative pedagogy to affect gains in social justice activism is minimal, although there is evidence that contemplative pedagogy can increase emotion regulation and improve participants’ levels of mental health symptoms and well-being (e.g., Waters et al., 2015). There is also meta-analytic evidence that mindfulness-based interventions can reduce prejudicial attitudes toward marginalized individuals (Chang et al., 2023).

Results from the tests of the long-run effects also suggest that a one-time intervention designed to target life purpose may affect gains in humility, albeit of shorter duration relative to gains in horizontal transcendence. A key element in life purpose interventions is the *interpersonal encounter*, and while Guerrero-Torrelles et al. (2017) emphasized the clinician – client encounter, connecting with others can also occur in the context of group interventions and relationships providing social support (Shin and Steger, 2014; Guerrero-Torrelles et al., 2017). Life purpose interventions also involve narrative strategies which can help individuals construct a coherent story around themes of life aims, agency, values, and self-positivity, designed to clarify “their unique niche within the world” (Shin and Steger, 2014, p. 95; Guerrero-Torrelles et al., 2017). *Life-crafting*, for example, may be a potentially useful narrative intervention, which involves having participants write about their best possible self in different areas of their lives, such as values, goals, and their envisioned future social life and career (Schippers and Ziegler, 2019). More recently, Schippers et al. (2023) offered a variation of life-crafting focused on writing about a best possible world, by imagining how changes to the world might improve peoples’ lives.

The notion of *niche* connotes awareness of self-in-context, or intersubjectivity, that is, the self in interdependent relation to the alterity of the other (Volf, 2019). Wargin (2022) contended that “the humble person is someone who has a proper perspective of their value in relation to others” (p. 57) and Spezio et al. (2019) noted that humility involves “openness to others, in which openness means the *inclusion of the other as valued together (inseparably) with the self”* (p. 6). These ideas seem to suggest that mindful awareness about self-in-relation may promote greater humility, as *interpersonal openness*. We contend that an intersubjective lens for the relation between life purpose and humility is distinct from a transcendence lens. Seeing oneself in context-bound interdependent relating is different than the more frequently posited frame of experiencing a sense of beyond-the-self connection to others.

The long-run effects examining impulse responses for humility on subsequent levels of humility were nonsignificant. This suggests that humility interventions may have little to no influence on later levels of humility, which is consistent with prior research showing that a spiritually-integrated workbook intervention designed to promote humility showed nonsignificant change in a sample of R/S leaders (Cuthbert et al., 2018). However, a humility intervention study with seminary students found that gains in humility were *concurrent* with gains in the capacities for self-regulation and relational flexibility (Jankowski et al., 2022d). The latter intersects with our current finding in that gains in humility may occur when other aspects of functioning are addressed. Future research could test a revised version of Jankowski et al.’s (2022d) group didactic intervention, with a self-participant designed practice component (e.g., self-care/rest), by integrating life purpose interventions.

Recent theorizing and research suggests that interventions designed to promote awe may affect gains in life purpose, horizontal transcendence, and humility (Ruberton et al., 2017; Thompson, 2022). Thompson (2022), for example, described a narrative mindfulness intervention indicating that reflecting on moments of a sense of vastness may foster meaning-making, felt connections to others, and humility. These interventions might be especially promising for R/S leaders, whose understandings of humility tend to emphasize relations with others *and* the divine (Wolfteich et al., 2021). Thompson’s (2022) awe intervention seems to belong to the larger class of savoring interventions, which have demonstrated effectiveness at reducing symptoms and increasing well-being (Smith et al., 2019; Carr et al., 2021). Smith et al. (2019) defined savoring as “amplifying or dampening positive emotions, as well as increasing or decreasing the duration of positive emotions” (p. 151). Smith et al. described the need to adapt savoring interventions to fit cultural context, for example, focusing on experiences of interpersonal connectedness and humility rather than self-focused positive emotions tied to celebrating moments involving personal benefit or reward and finding joy in personal accomplishments.

### Limitations and future directions

Our participants were adults obtaining Christian theological education in North America and did not represent all R/S identifications. As such, the generalizability of our findings for those identifying with other religions or reporting no religious identification may be limited. However, our sample demographics were comparable to a nationally representative US sample (Pew Research Center, 2018; Jankowski et al., 2022f). Further, while a majority of the sample reported Christian identification, approximately 15% reported “other” or “none,” with “other” in reference to four Christian categorizations and five world religions, suggesting the possibility of multi-religious identities. Based on a nationally representative US sample, 24% of the participants were considered multi-religious (Corcoran et al., 2021). Multi-, complex- (Corcoran et al., 2021), hybrid- (Ammerman, 2014) or pluralistic- (Bernhardt, 2017) religious identities consist of “draw[ing] on the spiritual sources of different religious traditions … amalgamated into a coherent whole” (Bernhardt, 2017, p. 415). Ammerman (2014) noted, “identities are always multistranded and intersectional” (p. 195), rather than “mutually exclusive (dis)belief categories” (Corcoran et al., 2021, p. 438). Examples of multi-religious self-identifications in the current study sample included Pentecostal protestant *and* Jewish, evangelical *and* mainline Protestant, and not identifying with fixed labels. Taken together, our sample of seminary students appears somewhat less representative of the larger North American population in terms of multi-and non-religious identifications, and yet, we believe there is some basis for generalizing the findings beyond our sample based on demographic comparisons with a representative US sample for age, gender, race, and ideological commitment (Pew Research Center, 2018; Jankowski et al., 2022f).

Future research should consider how to further refine our operationalization of religious affiliation and then model these multiple religious identifications. Berghuijs (2017), for example, recommended combining emic [i.e., “self-identification as a follower of a religion (I consider myself..;” p. 22) and etic (i.e., multi-dimensional assessment using items about affinity, practices, values, beliefs, participation)] operationalizations of religious identification. Berghuijs estimated that 23% of the Dutch population could be considered multi-religious. It may be that mixed method designs that incorporate qualitative data collection and analysis are needed to (a) generate quantitative variables that can tease out how religious identification influences or conditions the processes among virtues and well-being and/or (b) generate qualitative themes to contextualize quantitative findings.

Data collected after time 1 occurred post-pandemic declaration in the US, and it may be that the experience of the pandemic influenced the measurement of variables, and therefore the associations we observed. In particular, incidents of overt racism, heightened awareness of systemic racism, and political polarization during the pandemic in the US may have influenced participants’ responses to items in unaccounted for ways, especially those items about horizontal transcendence and social justice activism. Prior research conducted at time 1, delineated subgroups of participants in part by ideological commitment, social justice commitment, and marginalized sexual identification (Jankowski et al., 2022f). Specifically, Jankowski et al. (2022f) noted that one R/S profile seemed to “uniquely privilege autonomy and inquisitiveness” in relating to the sacred, “along with an activist ethic” (p. 673). In contrast, the reference subgroup was characterized by a controlled relating to the sacred, that is, “internally or externally pressured” (p. 672). This latter subgroup reported higher levels of virtues and well-being, and yet, simultaneously reported “greater exaggerated sense of relating to the sacred” (p. 669). They also reported more conservative ideology, lower social justice commitment, and sexual majority identification. Furthermore, Hydinger et al. (2017) found that more liberal ideological commitments predicted greater felt responsibility for reducing pain and suffering in the world. It seems likely therefore that distinct subgroups of participants may have responded to the pandemic differently.

The marginalized subgroup, for example, with more liberal ideology and greater social justice commitment may have been more motivated to engage in activism and may have experienced greater horizontal transcendence. The pandemic had a disproportionately negative influence on marginalized subgroups, typically exacerbating already existing difficulties (Schippers et al., 2022). Theoretically, conflict and suffering motivate horizontal transcendence and social justice activism (Wong et al., 2021 Barton and Hart, 2023), and research has documented the motivating influence of conflict and oppression on young adults’ civic engagement (Müller-Bachmann et al., 2023). In contrast, the controlled subgroup may be less likely to experience horizontal transcendence and engage in activism because of a lack of felt suffering. However, we did not find a difference in longitudinal associations comparing low and high levels of R/S interpersonal conflict. Nevertheless, we suspect that the larger social context likely shaped participants’ reactions to themes around civic engagement over the course of the pandemic in various ways. Future research should consider comparing longitudinal processes among horizontal transcendence and social justice activism with different subgroup categorizations for experiences of oppression, suffering, and/or injustice. Future research could also employ person-centered data analytic strategies to identify subgroups delineated by humility, civic engagement, and life purpose. In addition, future qualitative research is needed to describe participants’ experiences around themes related to horizontal transcendence and social justice activism in emic, nuanced ways.

There is also a growing literature on intellectual humility (IH) and R/S leaders (Choe et al., 2023), with one cross-sectional study with seminary students showing positive associations between IH and social justice activism (Paine et al., 2022). However, a cross-sectional study with undergraduate students found no association between IH and social justice commitment and civic engagement (Mcelroy-Heltzel et al., 2023). In addition, in a cross-sectional study with R/S leaders, greater IH predicted lower well-being through greater insecure attachment with God, when general humility was lower; and greater IH predicted lower grandiosity when religious exploration was high (Jankowski et al., 2019). It seems IH might cut in different directions for R/S leaders, such as leading to questioning religious beliefs but also promoting openness to diversity and social justice. These findings for IH merit future longitudinal research among emerging and established R/S leaders. In fact, future research could explore the associations between IH, civic engagement, and narcissism, given that R/S leaders may be susceptible to narcissism and the finding that greater IH corresponded to an indicator of greater vulnerable narcissism (Jankowski et al., 2019). In addition, Rico-Bordera et al. (2023) found a positive association between civic engagement and grandiose narcissism, suggesting a self-enhancement motive rather than the other-oriented motivation for activism.

We acknowledge the limitation of our conceptualization and modelling of “impulses as being akin to random assignment” (Zyphur et al., 2020a, p. 675), relative to experimental designs, and particularly experimental designs that examine the relative efficacy of actual interventions. However, as Zyphur et al. (2020a) noted, “even idealized methods such as randomized controlled trials (RCT) cannot enable unconditional inference because … the kind of relationships that they establish may be situated in contexts that do not help plan an intervention elsewhere” (p. 676). By comparison, the longitudinal panel design we employed using observational data has potential to “guide real-world action” by offering a level of external validity frequently not afforded by experimental designs (p. 676).

## Conclusion

We found robust evidence for a reciprocal influence between the presence of life purpose and horizontal transcendence, and long-run effects that implied that a random perturbation in the form of a one-time life purpose intervention may have an influence on later levels of horizontal transcendence. We also found evidence to suggest that a one-time social justice intervention may also influence later levels of horizontal transcendence and a life purpose intervention may influence later levels of humility.

## Data availability statement

The raw data supporting the conclusions of this article will be made available by the authors, without undue reservation.

## Ethics statement

The studies involving humans were approved by Institutional Review Board, Biola University. The studies were conducted in accordance with the local legislation and institutional requirements. The participants provided their written informed consent to participate in this study.

## Author contributions

SS: Funding acquisition, Writing – review & editing, Resources. DW: Funding acquisition, Project administration, Writing – review & editing, Investigation, Resources. MZ: Methodology, Writing – review & editing. SC: Writing – review & editing. EC: Writing – review & editing. PJ: Conceptualization, Data curation, Formal Analysis, Methodology, Writing – original draft, Writing – review & editing.
